# Reducing Hormonal Inputs in Rabbit Reproduction: Physical Ovulation Induction with a 3D-Printed Cannula

**DOI:** 10.3390/ani15172544

**Published:** 2025-08-29

**Authors:** Juan José Castillo, José Salvador Vicente, Francisco Marco-Jiménez, Enrique Aguilar, María Pilar Viudes-de-Castro

**Affiliations:** 1 Centro de Investigación y Tecnología Animal (CITA), Instituto Valenciano de Investigaciones Agrarias (IVIA), 12400 Segorbe, Castellón, Spain; castillo_juadia@gva.es; 2 Institute of Science and Animal Technology (ICTA), Universitat Politècnica de València, 46022 Valencia, Valencia, Spain; jvicent@dca.upv.es (J.S.V.); fmarco@dca.upv.es (F.M.-J.); 3 Centro de Agroingeniería, Instituto Valenciano de Investigaciones Agrarias (IVIA), 46113 Montcada, Valencia, Spain; aguilar_enrmar@gva.es

**Keywords:** rabbit, insemination, hormone-free, cannula stimulation

## Abstract

This study evaluated the effectiveness of a 3D-printed cannula in inducing ovulation in female rabbits without exogenous GnRH analogues. A total of 325 inseminations were performed across different physiological stages: nulliparous, multiparous lactating, and multiparous non-lactating does. Two insemination methods were compared: a physical stimulation technique using the 3D-printed cannula and a control method involving hormonal induction with buserelin acetate. Females were either synchronized with eCG or left untreated. Delivery rates and litter sizes were recorded to assess fertility outcomes. Results indicated that the insemination procedure had a noticeable effect on delivery rate, with the control group showing greater rates (79%) compared to the cannula group (65%). In conclusion, these findings suggest that physical stimulation via the cannula can induce ovulation without exogenous hormones. This hormone-free approach offers a promising and welfare-friendly alternative for rabbit artificial insemination, aligning with efforts to reduce hormone use in livestock production. Further optimization of cannula design and technique may improve results, particularly in less responsive groups, supporting sustainable and ethical reproductive management.

## Simple Summary

This study explored a more natural and animal-friendly way to help female rabbits ovulate (release eggs) during artificial insemination, without using synthetic hormones. Normally, rabbit breeders rely on hormone injections to trigger ovulation, but there is growing concern about their use in animal production due to animal welfare and environmental impact. The goal of this research was to test a specially designed tool—a 3D-printed insemination device—that mimics the physical stimulation of natural mating when inserted during insemination to see if it could trigger ovulation without hormones. Two methods were compared: the traditional hormone-based approach and this new hormone-free technique. A total of 325 inseminations were carried out on rabbits at different reproductive stages (young, lactating, and non-lactating adults). The results showed that while the hormone treatment group showed slightly higher birth rates overall, the hormone-free method still performed well—especially in lactating rabbits. In conclusion, this hormone-free approach could offer a safer, more sustainable, and welfare-friendly option for rabbit breeding. It supports better animal welfare and aligns with the growing demand for more sustainable and ethical farming practices. If refined further, it could help reduce hormone use in agriculture while maintaining productivity.

## 1. Introduction

Artificial insemination (AI) is a highly efficient assisted reproductive technology that has become a common practice in commercial rabbit farms, serving as the basis for modern rabbit production. This reproductive technology has improved rabbit breeding by enabling the development of advanced reproductive management systems, reducing the need for a large number of male breeders on farms, and allowing for the use of semen from genetically selected lines, positively impacting resource management and contributing to improved sanitary conditions within farms. Despite the many advancements in rabbit breeding made possible by AI, progress in the application of AI is still subject to physiological and technical limitations.

Rabbits are induced ovulators, an evolutionary adaptation that optimizes fertilization success by ensuring that ovulation occurs in close temporal proximity to mating [1]. In this species, the ovulatory process involves a neuroendocrine response that can be activated by various somatosensory stimuli associated with mating behaviour, including tactile, coital, visual, and olfactory cues [1,2,3,4]. In other induced ovulators, such as camelids, a seminal plasma-derived factor, specifically β-nerve growth factor (β-NGF), influences the activity of hypothalamic neurons that regulate GnRH secretion [5,6]. In rabbits, β-NGF has been identified in seminal plasma [7,8,9,10,11], and its receptors have been detected in the pituitary gland and reproductive tissues, including the ovaries, oviduct, and uterus [12,13]. Plasma β-NGF concentrations rise following natural mating or semen deposition [13], but, unlike in camelids, intramuscular injection of seminal plasma fails to induce an LH surge or ovulation [14,15]. Recombinant β-NGF alone also produces limited ovulation rates, even at supraphysiological doses [16,17], suggesting a supplementary rather than primary role in this species. Therefore, additional species-specific mechanisms are likely involved in triggering ovulation in rabbits.

Currently, ovulation during AI is achieved by administering GnRH or its analogues intramuscularly (0.8–1 micrograms/female). Although effective, animal welfare concerns have prompted the development of hormone-supplemented diluents that reduce handling stress and improve the biosafety of AI [9,18,19,20,21,22,23,24]. However, the vaginal bioavailability of GnRH analogues is low, generally below 20%, due to enzymatic degradation, tissue permeability, and diluent composition [8,22,23]. As a result, intravaginal doses must be 25–30 times higher than intramuscular doses to achieve comparable ovulation frequencies [24]. Strategies such as peptide encapsulation with chitosan, penetration enhancers, or aminopeptidase inhibitors have been proposed to improve GnRH absorption [8,24,25].

Adrenergic mechanisms may also play a role in ovulation induction. The stimulation of adrenergic receptors and catecholamine release modulate hypothalamic neuronal activity and pituitary secretion [26,27]. In rabbits, alpha-adrenergic receptors are present in the vaginal smooth muscle and blood vessels [28]. Contractile responses to adrenergic agonists vary throughout the vagina. The lower vaginal region primarily displays tonic responses, characterized by sustained and prolonged contractions, whereas the middle and upper vaginal regions predominantly exhibit phasic responses, marked by rapid and transient contractions [29]. Rich adrenergic innervation and small ganglia formations near the vaginal musculature have also been described [30]. These findings suggest that physical stimulation during insemination might activate neuroendocrine pathways involved in ovulation.

In addition to ongoing efforts to reduce the use of hormones in the ovulation induction of AI, non-hormonal strategies are also gaining interest for oestrus synchronization, essential for streamlining AI protocols and improving reproductive efficiency. The most used method involves the administration of equine chorionic gonadotrophin (eCG), which enhances female receptivity by promoting follicular development [31]. Temporary separation of lactating does from their litters has proven effective in mitigating the inhibitory effect of lactation on reproductive activity and stimulating sexual receptivity, while in non-lactating multiparous and nulliparous females, vulvar coloration provides a practical indicator of oestrus. These strategies offer promising hormone-free synchronization protocols in commercial rabbit production systems.

Previous studies have demonstrated that, in rabbit does synchronized with eCG, it is possible to induce ovulation solely through the introduction of an appropriate insemination cannula, achieving an ovulation frequency of 64% [32]. More recently, Viudes-de-Castro et al. [33] and Castillo et al. [34] reported an ovulation frequency of approximately 65% and delivery rates of 63% in nulliparous rabbits using a prototype cannula with multiple rings or adaptations of commercial cannulas. In contrast, this rate dropped to 35% and 30%, respectively, in multiparous rabbits. Additionally, Castillo et al. [35], using a double stimulation technique, where a commercial cannula was inserted twice to a depth of 15 cm at a 5 min interval, observed an ovulation frequency of 73% and a fertility rate at birth of 68% in nulliparous rabbits. These findings highlight the potential of using physical stimulation to trigger ovulation in female rabbits.

The present study aimed to evaluate the efficacy of a novel 3D-printed cannula for inducing ovulation in breeding females at different physiological stages (nulliparous, multiparous lactating, and multiparous non-lactating) without the use of GnRH analogues, using two synchronization protocols: eCG administration and non-hormonal methods.

## 2. Materials and Methods

Unless otherwise stated, all chemicals and reagents were purchased from Sigma-Aldrich (Merck Life Science S.L.U. Madrid, Spain).

### 2.1. Animals

The research was conducted at the experimental farm of the Institute of Science and Animal Technology (ICTA), “Universitat Politècnica de València”. Animals were handled in accordance with the principles of animal care outlined by European directive 2010/63/EU. All the experimental procedures employed in this study were reviewed and approved by the Ethical Committee for Animal Experimentation of the Universitat Politècnica de València, Spain (research code: 2024-VSC-PEA-0063).

Males and females were New Zealand White rabbits. All animals were housed individually in flat-deck cages under a 16 h light/8 h dark photoperiod, with temperatures maintained between 18 and 25 °C and humidity ranging from 60% to 85%. They were fed a standard pellet diet ad libitum (17.5% crude protein, 2.3% ether extract, 16.8% crude fibre, 2600 Kcal DE/Kg, NANTA S.A., Valencia, Spain) and provided with free access to water.

### 2.2. Vaginal Tissue Collection and Histological Processing

Six receptive New Zealand White rabbit does (three nulliparous and three multiparous, non-lactating) were euthanized. The reproductive tracts were dissected, and the vagina was isolated from the vulva to the cervix. The tissue was rinsed in phosphate-buffered saline (PBS), and samples from the proximal centimetre of the vaginal segment were collected. These samples were immediately fixed in 4% formaldehyde and subsequently processed for paraffin embedding. The fixed tissue was dehydrated through a graded ethanol series and embedded in paraffin. From each paraffin block, serial 5 µm-thick sections were obtained at 100 µm intervals and stained with haematoxylin and eosin. Histological sections were examined using an ZEISS Axioscope 5 microscope equipped with an Axiocam 208 colour camera (Carl Zeiss Microscopy GmbH, Oberkochen, Germany) for mechanoreceptors identification.

### 2.3. Semen Collection and Evaluation

Seminal samples were obtained from twelve males. Two ejaculates per male were collected on the same day using an artificial vagina, with a minimum interval of 30 min between collections. Ejaculates with a white colour, less than 15% abnormal sperm, and a motility rate above 70%, were pooled for use in the experiment; all others were discarded. An aliquot from each pooled sample was analyzed to assess semen quality.

A 20 μL aliquot was diluted 1:25 with 0.25% glutaraldehyde in phosphate-buffered saline solution to calculate sperm concentration in a Thoma chamber and to evaluate both the percentages of spermatozoa with normal apical ridge and abnormal sperm by phase-contrast microscopy at a magnification of 400×.

Sperm motility characteristics were evaluated following the protocol described by Viudes de Castro et al. [23], using a computer-assisted sperm analysis system (ISAS, version 1.0.17, Proiser, Valencia, Spain) operating at 30 frames per second (30 Hz) and configured to detect particles with an area between 20 and 80 μm^2^. Prior to analysis, sperm samples were diluted to a concentration of 14 million sperm/mL using a Tris-citric acid-glucose (TCG) extender, prepared as previously described [36], and supplemented with 2 g/L BSA. The diluted samples were then loaded into a Makler counting chamber (Sefi Medical Instruments, Haifa, Israel) and motility was assessed at 37 °C using negative phase contrast optics under 100× magnification with a NIKON Eclipse 90i microscope (Nikon Corporation Instruments Company; IZASA, Barcelona, Spain), connected to a computer via a monochrome Basler A312f video camera (Basler AG, Ahrensburg, Germany). At least four fields per sample were recorded. Sperm were classified as non-motile if their average path velocity (VAP) was below 10 μm/s, while those with a VAP greater than 50 μm/s and a straightness index above 70% were considered progressively motile. To ensure data accuracy, individual sperm tracks were visually reviewed to eliminate artefacts, debris, or incorrectly identified tracks.

Flow cytometric analyses to assess sperm viability (membrane integrity) and acrosome status were performed using a CytoFLEX flow cytometer (Beckman Coulter, Inc., Brea, CA, USA) equipped with red (638 nm), blue (488 nm), and violet (405 nm) lasers, operated with CytExpert Software v2.3. The cytometer was calibrated daily using specific calibration beads provided by the manufacturer. Data were collected from 10,000 events. Spermatozoa population was gated after Hoechst 33342 staining to eliminate non-sperm events. Doublets and cell clumps were excluded using a plot of side scatter area vs. side scatter height, followed by gating single events only. Compensation for spectral overlaps was performed before each experiment.

A triple staining method with FITC-PNA/PI/Hoechst, previously validated for rabbit semen in our laboratory, was used to evaluate viability and acrosomal integrity. For staining, 100 µL of semen at 30 × 10^6^ sperm/mL was incubated with 0.5 µL of Hoechst 33342 (0.5 mg/mL) for 20 min at 37 °C in the dark. Then, 1.5 µL of FITC-PNA (1 mg/mL) and 0.5 µL of PI (1 mg/mL) were added and incubated for 10 min at 37 °C, also protected from light. Afterwards, 400 µL of TCG extender was added to achieve a final concentration of 6 × 10^6^ sperm/mL. PI-negative sperm were considered viable. The percentage of sperm with a normal apical ridge was calculated as the proportion of sperm with intact acrosomes.

To prepare seminal doses, pooled semen was diluted to 40 million sperm/mL by adding TCG extender.

### 2.4. Canula Design

**Figure 1:**
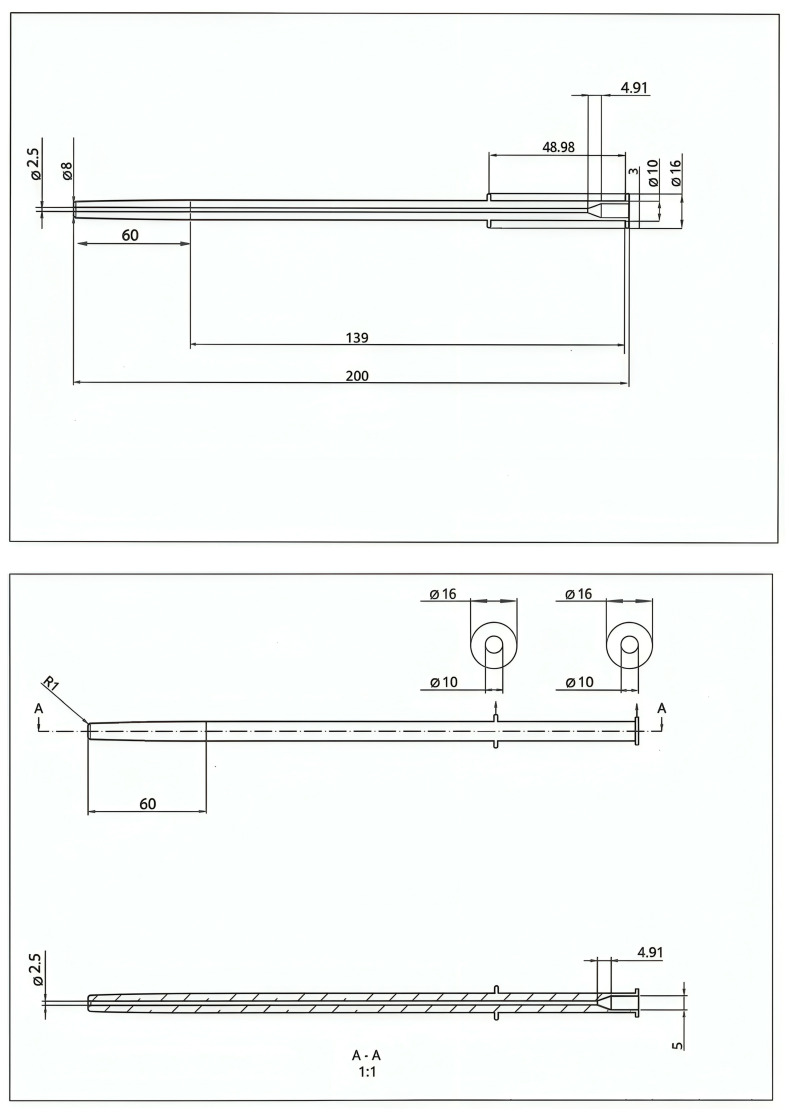
Insemination cannula design plans for 3D printing.

**Figure 2:**
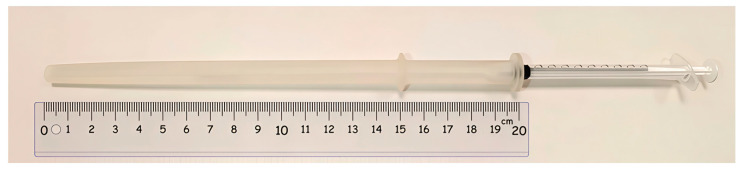
Flexible insemination cannula, 20 cm long and 10 mm thick, with ring stopper to limit the insertion depth to 15 cm, 3D printed.

The cannula prototype was designed considering the anatomical characteristics of the vagina and data from previous research on various cannula types [32,33,34,35]. The cannula was designed using Onshape CAD 3D software (Figure 1). Files were exported and saved in STL format for fabrication by 3D printing with Prusament Resin Flex80 Transparent Clear (Prusa Research a.s., Prague, Czech Republic) (Figure 2) (Tensile strength: 8.87 MPa, Elongation: 60.25%, Tensile modulus: 17.87 MPa, Viscosity at 25 °C: 200/400 mPa·s).

To control the insertion depth, the insemination cannula was designed with a 16 mm diameter ring, limiting penetration to 15 cm. The cannula itself has a thickness ranging from 8 to 10 mm. Compared to commercial models, the prototype is more flexible than polystyrene-based cannulas but more rigid than silicone ones. Additionally, its thickness exceeds that of standard commercial cannulas, which typically range from 5 to 6 mm in diameter.

### 2.5. Synchronization Receptivity Method

Two methods of receptivity synchronization were employed, and only receptive females were inseminated. Females were randomly assigned to one of the two receptivity synchronization groups: the eCG group, where 190 females were administered 12.5 IU of eCG intramuscularly two days before insemination (Cuniser500, Hipra, Girona, Spain), and the Bio group, in which females did not receive any hormonal treatment, and 135 receptive females (red colouration of vulvar lips) were inseminated. For lactating females, the nest was closed 36 h prior to insemination to ensure they were receptive and exhibited red vulvar colour at the time of insemination.

### 2.6. Insemination Procedure

Females were inseminated using a 3D-printed cannula prototype (Figure 1), with a total of 10 units produced. Each cannula was thoroughly cleaned and disinfected after every insemination to ensure hygiene and prevent cross-contamination. At the time of insemination, two insemination procedures were applied. In the first procedure (the cannula group), the cannula was inserted twice, with a 5 min interval between insertions [35]. The deposition of 0.5 mL of diluted semen was performed during the second insertion of the cannula. In the second procedure (the control group), females were inseminated using the same cannula but with a single insertion and treated intramuscularly with 1 µg of buserelin acetate (aGnRH, Hoechst Marion Roussel, S.A., Madrid, Spain).

A total of 325 inseminations were performed in females categorized by physiological status: nulliparous (n = 97; 69 cannula, 28 control) inseminated at 18–20 weeks; lactating multiparous (n = 116; 77 cannula, 39 control) inseminated 12 days postpartum with litters equalized to nine pups; and non-lactating multiparous (n = 112; 74 cannula, 38 control) inseminated after weaning.

Each physiological group was further divided according to the synchronization method used. Nulliparous and non-lactating multiparous does were either treated with eCG or received no hormonal treatment. In lactating multiparous does, synchronization was achieved either by eCG injection or through biostimulation via nest closure.

In all cases, insemination was performed only when females exhibited clear signs of receptivity (red vulvar coloration). Delivery rate and litter size at birth were recorded for each female.

### 2.7. Statistical Analysis

A General Linear Model (GLM) was used to evaluate mechanoreceptors in the vaginal epithelium and the total and live litter size at birth using the SPSS 23.0 software package (IBM SPSS Statistics for Windows, Version 23.0. Armonk, NY, USA: IBM Corp.). Delivery rate was analyzed using a probit link function with a binomial error distribution. In the model, the main factors of the insemination procedure (Control-aGnRH and cannula), the physiological state of females (nulliparous, multiparous lactating, and multiparous non-lactating females), synchronization treatment (eCG or Bio), and all their two-way interactions were included in the analysis. Shapiro–Wilk tests were conducted in the SPSS Explore procedure to assess the normality of the residuals (Gaussian distribution), and homogeneity of variances was evaluated through Levene testing. Differences between groups were assessed using Bonferroni’s test. Values were considered statistically significant at *p* < 0.05. Data are expressed as the least square mean ± standard error of the mean (LSM ± SEM).

## 3. Results

**Figure 3:**
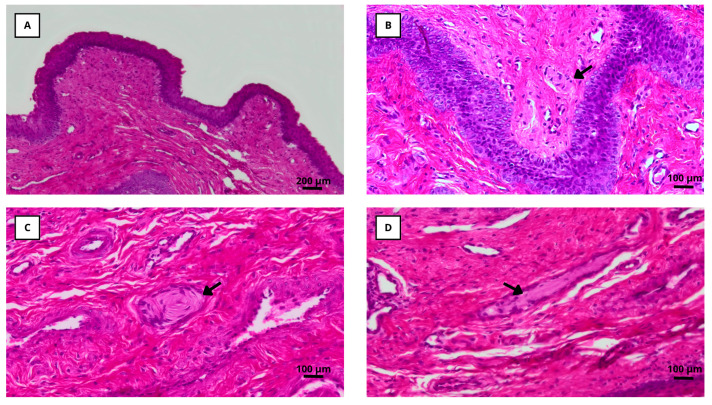
Histological sections of rabbit vaginal tissue. (**A**) Cross-section from the first centimetre of the rabbit vagina (100× magnification), showing the non-keratinized stratified squamous epithelium and lamina propria. (**B**) Meissner corpuscle (200× magnification). (**C**) Pacinian corpuscle (200× magnification). (**D**) Ruffini corpuscle (200× magnification).

Mechanoreceptors were identified in the vaginal epithelium of all examined specimens, predominantly localized in the basal and subepithelial layers. Based on their morphological characteristics under hematoxylin–eosin staining, they were classified as Meissner, Pacinian, or Ruffini corpuscles (Figure 3).

The total number of mechanoreceptors was significantly higher in nulliparous compared to multiparous does (8.9 ± 0.7 vs. 6.6 ± 0.7; *p* < 0.05, Table 1). While the counts of Meissner and Ruffini corpuscles did not differ significantly between groups, nulliparous does exhibited a higher number of Pacinian corpuscles than multiparous ones (3.97 ± 0.38 vs. 1.23 ± 0.37; *p* < 0.05).

The semen pools used in the experiment presented an average sperm concentration of 388 ± 22.2 × 10^6^ sperm/mL and contained 82.7 ± 7.72.0% total motile sperm, 82.6 ± 1.91% live sperm, 97.5 ± 0.62% sperm with a normal apical ridge, 4.7 ± 1.93% of sperm with cytoplasmatic droplets, and 89.1 ± 1.52% of normal sperm (data not presented in tables).

Among all the factors and two-way interactions analyzed for reproductive performance parameters, only the insemination procedure had a statistically significant effect on delivery rate (*p* < 0.05, Table 2). Neither the physiological status of the females, the synchronization method, nor any of the two-way interactions among the main factors showed significant effects on delivery rate, total litter size, or live litter size (Table 2 and Table 3).

However, a trend was observed in the interaction between the physiological status and insemination procedure on delivery rate (*p* < 0.1), suggesting a potential modulatory effect worth further investigation.

The delivery rate in females from the control group (induced to ovulate with buserelin acetate) was significantly higher than that in the cannula group (79 ± 4.2% vs. 65 ± 3.3%, respectively; *p* < 0.05, Table 2).

## 4. Discussion

The classification of mammals as either spontaneous or induced ovulators is based on the mechanism that triggers the release of GnRH and initiates the ovulatory cascade. In spontaneous ovulators, the preovulatory LH surge is initiated by the positive feedback mechanism of increasing circulating oestradiol levels in the absence of progesterone. In contrast, in induced ovulators, the primary trigger for the LH surge is a neuroendocrine response elicited by physical stimulation of the genitalia during copulation or other sensory stimuli. Rabbits are induced ovulators, requiring coitus to stimulate GnRH release [2,3].

However, induced ovulation is not a uniform phenomenon across species. Based on the duration and nature of the copulatory stimulus required, three categories have been proposed: short-duration copulators, such as rabbits, in which a single brief mating suffices; prolonged-duration copulators, such as cats and ferrets, requiring multiple or extended matings, and seminal plasma-mediated induced ovulators, such as camelids, where ovulation depends primarily on seminal factors rather than mechanical stimulation [1,4,14,37]. These categories highlight the diversity of strategies that ensure reproductive success in induced ovulators and underline the importance of species-specific strategies in reproductive physiology.

From a practical perspective, in the context of AI in rabbits, the absence of copulation has traditionally required ovulation induction with exogenous GnRH analogues. However, growing concerns about sustainability and animal welfare have spurred interest in developing hormone-free alternatives. An innovative approach aims to mimic the physical stimulation that occurs during natural mating. Earlier studies have demonstrated that ovulation could be triggered in 64% of does by using a specially designed insemination cannula [32]. More recently, Viudes-de-Castro et al. [33] used a 3D-printed cannula with specific dimensions and stimulation rings, yielding ovulation and parturition rates of 65% and 63% in nulliparous does, while the corresponding rates in multiparous does were notably lower (35% and 30%), suggesting that physiological differences between these reproductive states may modulate the effectiveness of physical stimulation.

Encapsulated mechanoreceptors, particularly Pacinian corpuscles, may explain part of this variation. These receptors detect high-frequency vibrations and pressure and are well preserved in nulliparous females, whereas parity-related hormonal and mechanical changes may reduce their density and function [38,39]. Their abundance in nulliparous does likely enhances the neuroendocrine reflexes and muscle contractions required for ovulation [29,40,41], while their reduction in multiparous females could limit responsiveness to mechanical stimulation. Repeated parturitions alter pelvic muscle contractility pressure dynamics, and ganglionic architecture, potentially affecting the neuroendocrine response to mechanical stimulation [42,43,44,45,46].

These mechanistic insights into parity-related changes in vaginal mechanoreceptors and neuromuscular responses provide a strong rationale for adapting stimulation protocols to each reproductive status. Castillo et al. [35] showed that a double-stimulation protocol improved ovulation rates from 55% to 73%. In our current work, increasing cannula thickness and combining it with double-insertion strategy enhanced outcomes in multiparous non-lactating does and even yielded viable results in lactating ones, a traditionally less responsive group. By contrast, in nulliparous does, this combination may have induced excessive mechanical stress, slightly lowering fertility compared with previous results [34,35].

Anatomical data reinforces this interpretation. The rabbit vagina contains dense adrenergic innervation and ganglia in the mid vaginal region [30], suggesting that increasing cannula diameter and depth of insertion could potentiate afferent signalling and GnRH release. However, further studies combining histological and endocrine data are needed to confirm this hypothesis.

## 5. Conclusions

This study demonstrates that physical stimulation protocols tailored to reproductive status can effectively induce ovulation in rabbits without the use of exogenous hormones. The use of a larger-diameter insemination cannula, combined with double stimulation, clearly improves ovulation and fertility outcomes in multiparous females by compensating for parity-related reductions in mechanoreceptor density and pelvic muscle responsiveness. Conversely, in nulliparous does, the same strategy may lead to receptor overstimulation, which could explain the lower fertility rates observed compared with previous studies.

These findings provide a physiological basis for optimizing device design and stimulation patterns based on reproductive status, and could further enhance reproductive efficiency and support a shift toward more sustainable and welfare-oriented rabbit production. Future research should focus on confirming the histological changes associated with parity, refining stimulation protocols, and integrating this approach with other non-hormonal management strategies to further improve reproductive efficiency.

## Figures and Tables

**Table 1:** Number of mechanoreceptor types identified in the vaginal epithelium of nulliparous and multiparous receptive females.

	Meissner Corpuscles LSM ± SEM	Pacinian Corpuscles LSM ± SEM	Ruffini Corpuscles LSM ± SEM	Total LSM ± SEM (n)
**Multiparous**	1.0 ± 0.16	1.2 ± 0.38 ^a^	4.4 ± 0.44	6.6 ± 0.68 ^a^ (30)
**Nulliparous**	0.6 ± 0.16	4.0 ± 0.38 ^b^	4.3 ± 0.44	8.9 ± 0.68 ^b^ (30)
*p*-Value	0.078	<0.001	0.929	0.046
**Total**	0.8 ± 0.11	2.6 ± 0.26	4.3 ± 0.31	7.7 ± 0.48

LSM ± SEM (Least Square Mean ± standard error of the mean). ^a,b^ Values with different superscripts in the same column differ significantly (*p* < 0.05). (*n*) Number of examined specimens.

**Table 2:** Reproductive performance at birth by main factors (insemination procedure, physiological status, and synchronization treatment).

Factors		Delivery Rate LSM ± SEM	Total Litter Size LSM ± SEM	Live Litter Size LSM ± SEM
**Insemination procedure**	Control (*n*)	79 ± 4.2 ^a^ (105)	10.3 ± 0.30 (80)	10.1 ± 0.34 (80)
	Cannula (*n*)	65 ± 3.3 ^b^ (220)	10.5 ± 0.23 (145)	9.9 ± 0.26 (145)
*p*-Value		0.011	0.723	0.562
**Physiological status**	Nulliparous (*n*)	76 ± 5.2 (97)	10.4 ± 0.34 (68)	9.9 ± 0.38 (68)
	Multiparous lactating (*n*)	66 ± 4.8 (116)	10.8 ± 0.33 (76)	10.7 ± 0.37 (76)
	Multiparous non-lactating (*n*)	75 ± 4.6 (112)	9.9 ± 0.32 (78)	9.5 ± 0.36 (78)
*p*-Value		0.294	0.152	0.071
**Synchronization**	eCG (*n*)	72 ± 3.7 (190)	10.3 ± 0.25 (134)	10.0 ± 0.29 (134)
	Bio (*n*)	73 ± 4.3 (135)	10.5 ± 0.29 (91)	10.0 ± 0.32 (91)
*p*-Value		0.988	0.662	0.970
**Total** **(*n*)**		73 ± 2.9 (325)	10.4 ± 0.19 (225)	10.0 ± 0.21 (225)

Control: one cannula insertion and 1 μg of busereline acetate intramuscularly. Cannula: double cannula insertion. eCG: females synchronized with eCG Bio: Females that have not received hormone synchronization treatment. LSM ± SEM (Least Square Mean ± standard error of the mean). ^a,b^ Values with different superscripts in the same row or column differ significantly (*p* < 0.05). (*n*) Number of inseminated does.

**Table 3:** Reproductive performance of two-way interaction of physiological status, synchronization treatment, and insemination procedure.

Physiological Status by Insemination Procedure			Delivery Rate LSM ± SEM	Total Litter Size LSM ± SEM	Live Litter Size LSM ± SEM
	Nulliparous	Control (*n*)	86 ± 6.6 (28)	10.0 ± 0.55 (24)	9.5 ± 0.62 (24)
		Cannula (*n*)	64 ± 5.8 (69)	10.8 ± 0.41 (44)	10.3 ± 0.45 (44)
	Multiparous lactating	Control (*n*)	65 ± 7.7 (39)	11.2 ± 0.54 (25)	11.2 ± 0.60 (25)
		Cannula (*n*)	68 ± 5.6 (77)	10.5 ± 0.39 (53)	10.2 ± 0.43 (53)
	Multiparous non-lactating	Control (*n*)	84 ± 5.9 (38)	9.8 ± 0.49 (31)	9.7 ± 0.55 (31)
		Cannula (*n*)	64 ± 5.8 (74)	10.1 ± 0.41 (48)	9.3 ± 0.45 (48)
*p*-Value			0.070	0.266	0.199
**Synchronization treatment by insemination procedure**					
	eCG	Control (*n*)	77 ± 5.8 (58)	10.3 ± 0.42 (43)	10.3 ± 0.47 (43)
		Cannula (*n*)	68 ± 4.1 (132)	10.3 ± 0.29 (91)	9.8 ± 0.32 (91)
	Bio	Control (*n*)	82 ± 5.7 (47)	10.4 ± 0.44 (37)	10.0 ± 0.49 (37)
		Cannula (*n*)	61 ± 5.2 (88)	10.6 ± 0.37 (54)	10.1 ± 0.41 (54)
*p*-Value			0.241	0.710	0.577
**Physiological status by synchronization treatment**					
	Nulliparous	eCG (*n*)	75 ± 7.0 (47)	10.8 ± 0.50 (33)	10.4 ± 0.55 (33)
		Bio (*n*)	78 ± 6.7 (50)	10.0 ± 0.46 (35)	9.4 ± 0.51 (35)
	Multiparous lactating	eCG (*n*)	66 ± 5.9 (74)	10.2 ± 0.41 (50)	10.2 ± 0.46 (50)
		Bio (*n*)	67 ± 7.4 (42)	11.5 ± 0.51 (28)	11.1 ± 0.57 (28)
	Multiparous non-lactating	eCG (*n*)	77 ± 5.5 (69)	9.9 ± 0.39 (51)	9.5 ± 0.43 (51)
		Bio (*n*)	73 ± 7.2 (43)	9.9 ± 0.51 (28)	9.5 ± 0.57 (28)
*p*-Value			0.787	0.070	0.176

Control: one cannula insertion and 1 μg of busereline acetate intramuscularly. Cannula: double cannula insertion. eCG: females synchronized with eCG. Bio: Females that have not received hormone synchronization treatment. LSM ± SEM (Least Square Mean ± standard error of the mean). (*n*) Number of inseminated does.

## Data Availability

The original contributions presented in this study are included in the article, and further inquiries can be directed to the corresponding authors.

## Abbreviations

The following abbreviations are used in this manuscript:

AI	Artificial insemination
GnRH	Gonadotropin-releasing hormone
eCG	Equine chorionic gonadotropin
LH	Luteinizing hormone
β-NGF	β-Nerve growth factor
TCG	Tris-citric acid-glucose
FITC-PNA	FITC-labelled peanut agglutinin
PI	Propidium iodide
LSM	Least square mean
SEM	Standard error of the mean
GLM	General linear model
